# Ocean acidification compromises a planktic calcifier with implications for global carbon cycling

**DOI:** 10.1038/s41598-017-01530-9

**Published:** 2017-05-22

**Authors:** Catherine V. Davis, Emily B. Rivest, Tessa M. Hill, Brian Gaylord, Ann D. Russell, Eric Sanford

**Affiliations:** 1 Department of Earth & Planetary Sciences, University of California - Davis, Davis, CA USA; 2 Bodega Marine Laboratory, University of California - Davis, Bodega Bay, CA USA; 3 Department of Evolution and Ecology, University of California - Davis, Davis, CA USA; 4 Virginia Institute of Marine Science, William & Mary, Gloucester Point, VA USA

## Abstract

Anthropogenically-forced changes in ocean chemistry at both the global and regional scale have the potential to negatively impact calcifying plankton, which play a key role in ecosystem functioning and marine carbon cycling. We cultured a globally important calcifying marine plankter (the foraminifer, *Globigerina bulloides*) under an ecologically relevant range of seawater pH (7.5 to 8.3 total scale). Multiple metrics of calcification and physiological performance varied with pH. At pH > 8.0, increased calcification occurred without a concomitant rise in respiration rates. However, as pH declined from 8.0 to 7.5, calcification and oxygen consumption both decreased, suggesting a reduced ability to precipitate shell material accompanied by metabolic depression. Repair of spines, important for both buoyancy and feeding, was also reduced at pH < 7.7. The dependence of calcification, respiration, and spine repair on seawater pH suggests that foraminifera will likely be challenged by future ocean conditions. Furthermore, the nature of these effects has the potential to actuate changes in vertical transport of organic and inorganic carbon, perturbing feedbacks to regional and global marine carbon cycling. The biological impacts of seawater pH have additional, important implications for the use of foraminifera as paleoceanographic indicators.

## Introduction

Global change, driven by the anthropogenic release of carbon dioxide (CO_2_) into the atmosphere, is rapidly altering modern oceans. The oceans have already absorbed a third of emitted anthropogenic CO_2_^1^, and the resulting decrease in ocean pH and carbonate saturation state, known as ‘ocean acidification’, will impact the physiology, ecology, and preservation of marine fauna (e.g. refs 2–5). Biological responses to ocean acidification vary by taxon^5–7^ and by geographic region^8–10^. Among pelagic calcifiers, reductions in pH and concentration of carbonate ion (CO_3_^2−^) are known to alter the performance of pteropods^11–13^, coccolithophores^14–16^, and foraminifera^17–20^. These marine calcifiers are ubiquitous in the world’s oceans, where they play essential roles in food web dynamics and carbon cycling, and provide vital tools for paleoclimate research.

Along the California margin in the northeastern Pacific Ocean, planktic foraminifera affect carbon flux in two ways. First, their shells, together with those of coccoliths, contribute CaCO_3_ to the ‘alkalinity pump’, which transports inorganic carbon out of surface waters while reducing alkalinity (a potential positive feedback to climate change and amplification of surface acidification^21, 22^). Indeed, the shells of these two groups comprise 20–80% of marine calcite exported to the deep ocean^21, 22^. Second, inorganic and organic components of vertical carbon transport interact such that shell material may act as “ballast” for relatively light and unstructured organic matter. In this regard, foraminiferal shell properties may control sinking rates of soft tissue and the efficiency of the biologic carbon pump, a negative feedback on climate change and mitigation of surface ocean acidification^23–28^. Beyond their role in carbon cycling, planktic foraminifera are excellent archives of the water column conditions in which they calcify during their short lives. These organisms are therefore valued for their rich fossil record, which facilitates reconstruction of past ocean conditions (e.g. refs 29, 30).

At present, knowledge concerning the response of foraminifera to changes in ocean chemistry remains incomplete. While reductions in planktic foraminiferal shell weight have been observed under decreased seawater pH in the laboratory and in some field studies^18–21, 31^, other studies show no such trend^32, 33^. Where observed, correlations between pH and shell weight have been attributed to increases in the energy requirements of calcification under low pH conditions^34, 35^. However, additional physiological consequences of ocean acidification, including maintenance of key body structures that can influence net buoyancy and feeding, and overall reproduction and metabolic functioning – all of which can affect mortality, rates of sinking, and ultimately carbon export – have not been examined.

Here, we provide a first exploration of additional biological consequences of ocean acidification by culturing the widespread planktic foraminifer, *Globigerina bulloides*, a common focal taxon in paleoceanographic research, under a range of seawater pH. This species complex, with 7 known genetically distinct lineages, has a cosmopolitan distribution and ranges from polar regions to the tropics, including both upwelling zones and oligotrophic gyres^36–38^. Within the California Current Upwelling System, these organisms already experience a wide range of environmental pH, encompassing minima that are expected to intensify and increase in frequency in the coming century, with potential consequences for carbon cycling and export^39, 40^. Therefore, we examine several types of responses of *G*. *bulloides* to increased ocean acidity, including changes in calcification, morphology, and physiology. We follow these experiments with an initial evaluation of the implications of this suite of responses for survival of these organisms and the operation of both the biologic carbon and alkalinity pumps, as well as for the geologic record.

## Methods

### Collection

Individual *G*. *bulloides* were collected by 155 μm mesh net tows taken offshore of Bodega Head, CA in January-February 2015. Foraminifera were isolated from tow material and randomly assigned to a pH treatment into which they were placed immediately. Based upon the location of our study site, these *G*. *bulloides* likely belong to type IId or IIe as distinguished by past genetic studies^38^. Treatment seawater was filtered at 0.6 μm, before being chemically brought to a target pH at constant alkalinity^41^, and labeled with a fluorescent calcein dye. All foraminifera were held at 16 °C (±0.15 °C) under a stable 12 hour light/dark cycle, although *G*. *bulloides* lacks photosymbionts.

### Experimental Design

On day 2, after 24 hours recovery, individuals were fed a day-old freeze-killed *Artemia* nauplius. Foraminifera that fed successfully and had colored cytoplasm and active rhizopodia were designated with specimen numbers and tracked through the next phase of experimental observations. Treatment water in the culture vials was then replaced with fresh calcein-labeled treatment water. On day 3, 24 hours after feeding, 10–12 foraminifera per treatment were selected at random for oxygen consumption measurements and were triple-rinsed in filtered seawater to remove any calcein. Following respirometry trials, foraminifera were fed and returned to freshly poured calcein-labeled treatment water. Foraminifera were then observed every day and fed and imaged every other day with an accompanying water change, until death or gametogenesis.

### Respirometry

Individual foraminifera were placed in ~1 mL gas-tight biological oxygen demand (BOD) vials and sealed without headspace for 24 hours in darkness at 16 °C, along with two control vials containing only treatment water. After 24 hours, ~700 μL of seawater from each BOD vial was injected into a glass measurement cell, and the final oxygen concentration was read in triplicate using an optode, calibrated using a saturated solution of sodium sulfite (0% O_2_) and air-saturated seawater (100% O_2_). Oxygen consumption over the 24-hour incubation (nmol O_2_ foram^−1^ hr^−1^) was calculated with respect to the control vials.

### Fluorescence

Total calcite added was assessed by determining average pixel intensity of the visible shell. Incorporated calcein was excited and imaged at a wavelength of 495 nm using an epifluorescent AZ100 microscope with a standardized exposure time of 1.5 s. Average pixel intensity, a direct measure of calcein uptake into the shell during culture, was determined from the images using MetaMorph (Molecular Devices). Comparing average pixel intensity among treatment groups provides a relative quantification of the effects of seawater carbonate chemistry on shell calcification. See supplement for further details.

## Results and Discussion

**Figure 1:**
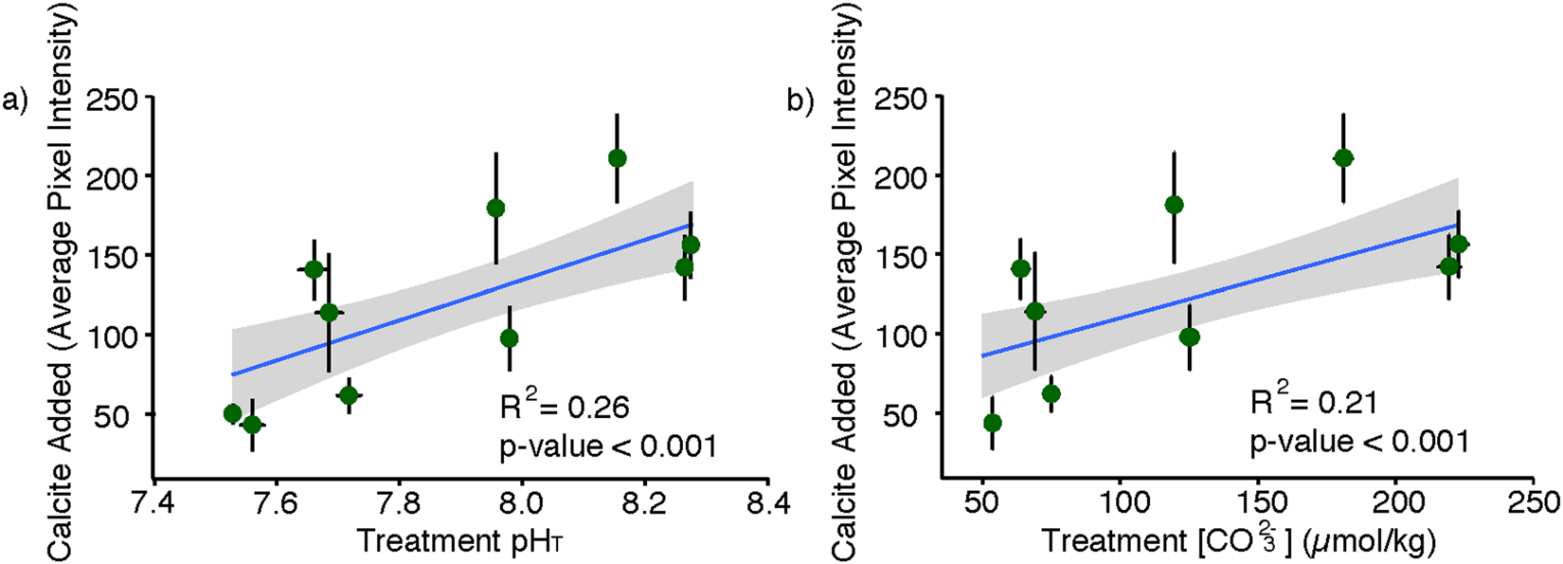
Mean calcification in *Globigerina bulloides* as measured by calcein uptake (average pixel intensity), with respect to (**a**) pH and (**b**) [CO_3_^2−^]. Error bars show standard deviation in both the x and y directions. These data are fit with a linear model with a 95% confidence envelope. The R^2^ and p-value refer to the fit of individual foraminifera to this curve (data for individual foraminifera can be found in Supplementary Data).

When *G*. *bulloides* was exposed to lower seawater pH, shell calcification was reduced (Fig. 1). Using an approach based on the incorporation of a fluorescent dye (calcein) into calcium carbonate shells, we documented a significant positive correlation between the amount of calcite added and pH (total scale) (p-value < 0.01; R^2^ = 0.26; Fig. 1a; Fig. S1A). Due to an increase in the calcite saturation state, a similar trend arose between calcification and carbonate ion concentration, [CO_3_^2−^] (p-value < 0.01; R^2^ = 0.21; Fig. 1b; Fig. S1B). These patterns concurred with results from previous culture studies, including one concerning the planktic foraminifera, *Orbulina universa* ^20, 31^, which showed a linear decrease in shell weight over a similar range of [CO_3_^2−^]. While a reduction in [CO_3_^2−^] over the range shown here would negatively impact foraminiferal calcification at the population level, the amount of CaCO_3_ added to the shell varied among individuals (Fig. S1B). If this variability in performance has a heritable genetic basis, natural selection may play an important role as a mechanism for increasing tolerance in foraminifera populations to decreasing ocean pH, as has been suggested in other calcifiers^42, 43^. In the absence of such genetic adaptation, the documented reductions in calcification indicate decreased overall mass density (less shell per body volume), and thus decreased contribution to inorganic carbon export as ocean acidification progresses.

**Figure 2:**
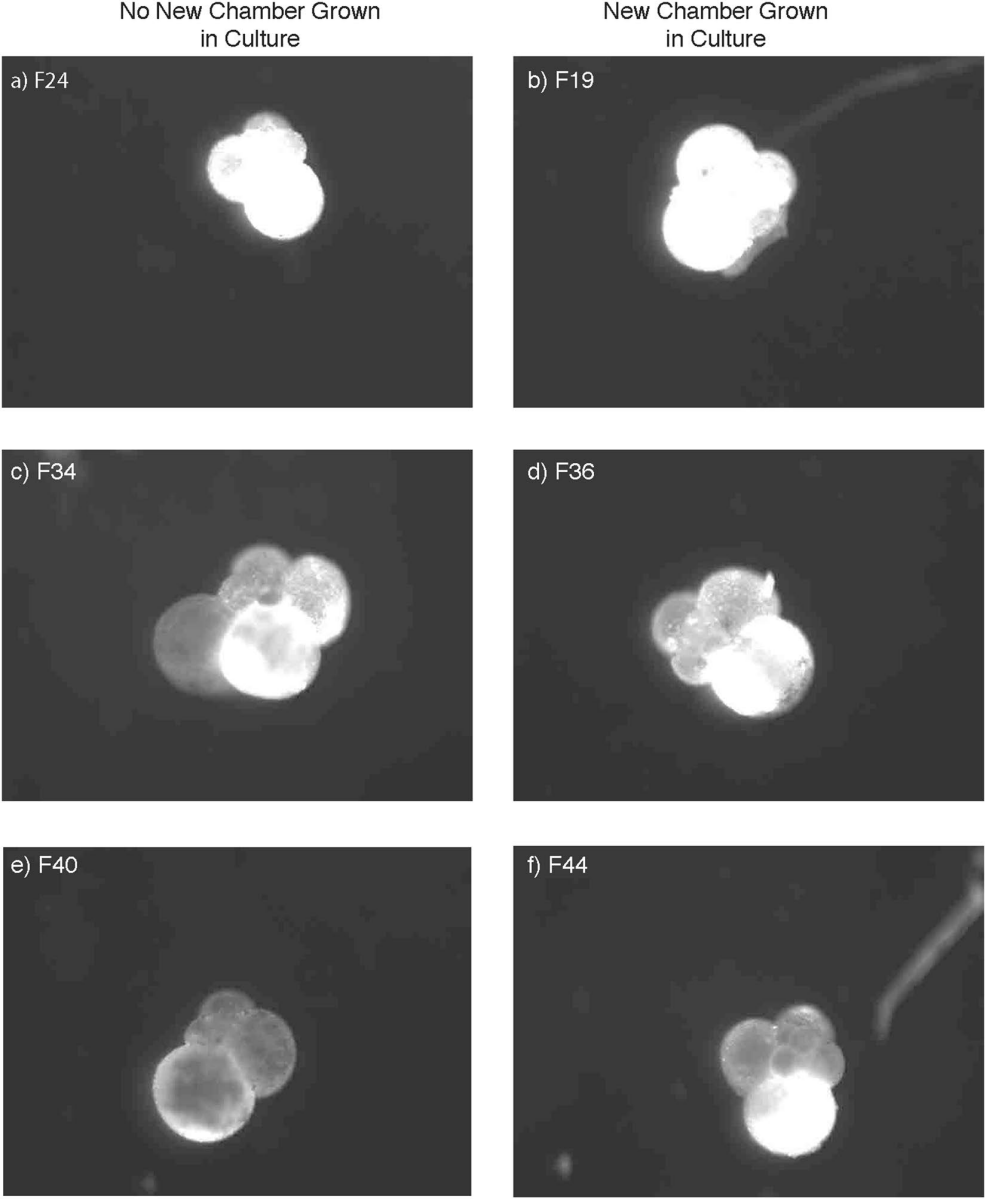
Patterns of calcein incorporation from *Globigerina* *bulloides* in culture. Several examples of the different patterns in shell calcification seen from foraminifera collected on the same date. Individuals F24 (**a**), F34 (**c**), and F40 (**e**) did not form a chamber in culture, although calcification occurred over the entire shell in F24, was focused on select chambers in F34 and was minimal in F40. Foraminifera F19 (**b**), F36 (**d**), and F44 (**f**) all grew a chamber with F19 showing calcification in both the new chamber as well as over the rest of the shell, F36 calcified most heavily in the new chamber and less over the rest of the shell, and F44 calcified primarily in the new chamber.

Planktic foraminifera are encapsulated by a calcite shell, which grows both by sequential addition of chambers and by thickening over pre-existing chambers^34, 43–45^ and many species, including *G*. *bulloides*, also possess calcite spines that extend outward from the shell wall. Interestingly, we observed a decoupling between chamber formation and addition of new calcite layers onto pre-existing chambers. Some foraminifera in our low-pH treatments added new chambers without adding detectible calcite onto older chambers. In contrast, in higher pH treatments, calcite was usually added over multiple existing chambers, including in individuals that did not form a new chamber in culture (Fig. 2). These observations differ from a common conceptual model in which the production of each new chamber occurs concomitantly with the addition of a new layer of calcite on all older chambers^43, 44^. For some individuals, the majority of calcification was concentrated in the most recent chamber, while in others, chamber formation was accompanied by detectable shell thickening on all chambers (Fig. 2). Furthermore, there were no significant differences across treatments in the proportion of foraminifera that added a new chamber in culture. Thus, chamber formation, rather than concurrent thickening, seems to be conserved during periods of stress, suggesting that it may be more essential for organismal functioning (e.g. reaching milestones such as attaining a minimal size for reproduction or accommodating cytoplasmic growth).

**Figure 3:**
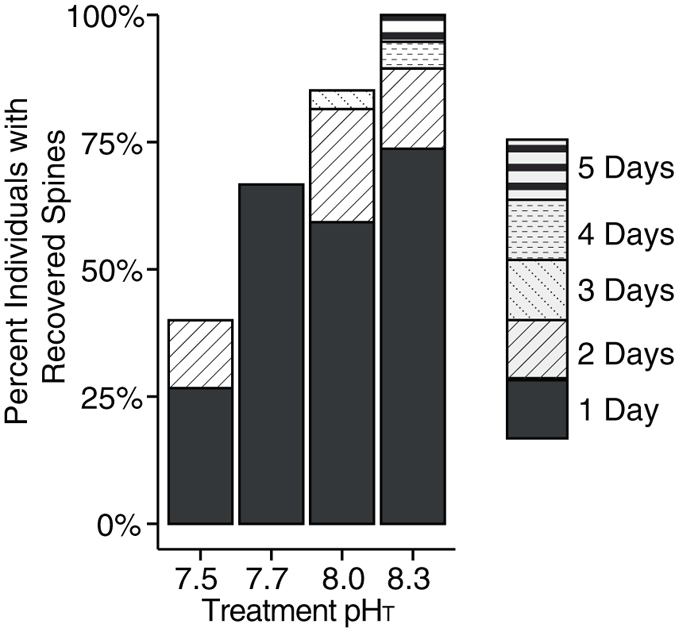
Percentage of individual foraminifera that regrew spines at each of four pH conditions. The number of days between experiment start and regrowth are shown in patterned bars. After 5 days, significant differences were found between foraminifera exposed to pH 7.5 vs. pH 8.0 and 8.3 (ANOVA; F_3,83_ = 13.64; p-value < 0.01).

Growth and maintenance of spines declined at lower pH (Fig. 3). In the water column, *G*. *bulloides* is surrounded by an array of thin rounded spines. Although the function of spines remains poorly understood, previous researchers have proposed that the spines are likely utilized in the capture and retention of live prey; spines will also act to slow sinking rates and therefore may act to maintain preferred vertical positioning in the water column^46, 47^. Whereas spinose foraminifera (including *G*. *bulloides*) drop their spines due to shock or mechanical damage during collection, lost spines are usually regenerated in culture within 1–2 days^46^. Complete spine recovery was observed in the highest pH treatments, in which all *G*. *bulloides* eventually regrew their spines, with longer recovery times of four and five days observed for some individuals. In marked contrast, only 30% of foraminifera cultured in pH group 7.5 regrew and maintained their spines (Fig. 3), a significant reduction from that of higher pH groups (F_3,83_ = 13.64; p < 0.01; Fig. 3). Foraminifera lacking spines may experience reduced buoyancy and/or difficulty capturing prey, and might therefore experience elevated non-reproductive mortality. Although foraminifera complete their life cycle by converting most of their tissue to gametes that are then expelled into shallow waters, individuals that die and sink prematurely would do so with soft tissue intact, which is ballasted by the shell itself. As spineless shells sink faster than shells with spines^48^, this non-reproductive mortality would enhance transport of foraminiferal organic carbon to depth.

**Figure 4:**
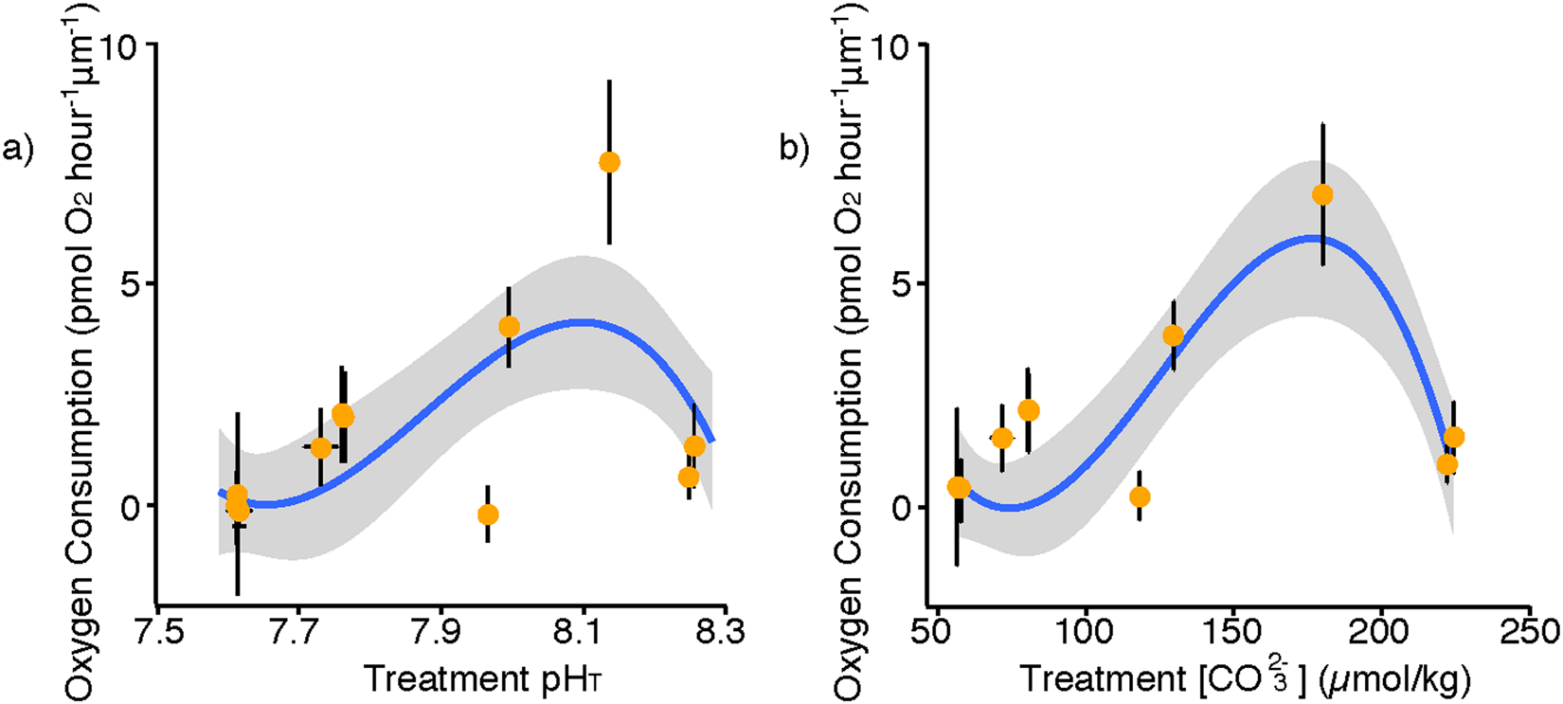
Oxygen consumption of *Globigerina bulloides* in response to changing carbonate chemistry conditions. Average rate of oxygen consumption for foraminifera over 24 hours, normalized to the longest shell diameter relative to the (**a**) pH and (**b**) [CO_3_^2−^] of exposure conditions (data for individual foraminifera can be found in Supplementary Data). Error bars show standard deviation in both the x and y directions. Data are fit with a 3^rd^ order polynomial with a 95% confidence envelope.

Respiration rates of foraminifera incubated under the lowest pH conditions were reduced to rates indistinguishable from background microbial respiration (Fig. 4; Fig. S2). Such low respiration rates are indicative of metabolic depression, a common response of marine invertebrates to physiological stress in which the organism minimizes energy-consuming processes, stalling growth and reproduction, in an effort to survive transient episodes of environmental stress. A range of respiration rates was observed in each experimental run varying from indistinguishable from background (<0.5 nmol O_2_ foram^−1^ hr^−1^) to 3.5 nmol O_2_ foram^−1^ hr^−1^ (Fig. S3). Average oxygen consumption increased with pH up to an inflection point at pH 8.1 ([CO_3_^2−^] 180 μmol kg^−1^), although with a large spread in individual respiration rates not explained by observed variables (Fig. S4). The pH conditions associated with maximum average respiration rates coincide with near surface conditions at collection, and thus it is possible that prior acclimatization to *in situ* pH may play some role in shaping the measured physiological responses. However, the observed variance in metabolism was not found to be significantly related to the absolute difference between carbonate chemistry conditions at collection and laboratory exposure (Supplementary Table S1). Thus acclimatization cannot be a primary driver for the observed response in oxygen consumption across the range of pH conditions.

The hypothesis that formation or maintenance of elevated pH vacuoles within the cytoplasm is necessary for foraminiferal calcification leads to the prediction that greater energy expenditure is required under low pH conditions^34, 49^. If the existing physiological capacity of *G*. *bulloides* for aerobic metabolism can accommodate increased energy demands under low seawater pH, rates of oxygen consumption would be expected to increase with decreasing pH. However, oxygen consumption declined with pH < 8.1, suggesting that conditions of ocean acidification may elicit metabolic depression in this population. Metabolic depression is an effective adaptive strategy for tolerating short-term environmental stress^50^. Under metabolic depression, energy turnover is decreased in order to ensure short-term survival of the organism; reproduction and growth are delayed or sacrificed, with implications for population dynamics^50, 51^. Signs of metabolic depression at pH < 7.7 indicate that satisfying any increase in demand for energy under acidified conditions was beyond the metabolic scope of the foraminifera. While metabolic depression may allow *G*. *bulloides* to tolerate short-term perturbations of low seawater pH (for example, upwelling events), individual fitness may be reduced if this species exhibits regulated reduction in metabolism to future long-term ocean acidification. Thus, a response of metabolic depression to long-term acidification would likely act in concert with reduced spine repair to increase non-reproductive mortality and organic carbon export.

At pH > 8.1, respiration rates of *G*. *bulloides* decreased (Figs 1 and 4). In contrast to low pH, respiration rates remained above background levels, while foraminifera added more calcite, demonstrated complete spine recovery, and performed well according to qualitative metrics including rhizopodial extent and cytoplasm color. Under high pH conditions, *G*. *bulloides* may require less energy expenditure to achieve high calcification rates. This could be due to lower acid-base regulation needed to maintain favorable conditions at the sites of calcification, or a higher proton gradient between the organism and the environment^52^. As a result, calcification may be less dependent on the rate of aerobic metabolism, and foraminifera may be able to precipitate more calcite with the same or even a reduced portion of their energy budget. In other protozoans, respiration rate has been closely linked to cell growth rate^53^. If the energetics of calcareous foraminifera are similar to those of naked ciliates, respiration rate may be considered representative of cell growth rate. Calcification may thus be energetically favored by any increase in environmental pH or [CO_3_^2−^] (over the range studied), while maximum cell growth rates are restricted to a narrower range of pH, reflected in rates of maximum respiration. Overall, the decoupling of respiration rate and total calcification observed at the highest pH treatment suggests that an increase in aerobic metabolism is not required to support increased calcification at higher pH.

A more complete picture of the relationship between seawater chemistry and foraminiferal calcification and physiology can improve predictions of how carbon cycling will be altered in an acidifying ocean. Foraminifera play an important role in ocean carbon cycling with a small but significant global organic carbon biomass of 0.0009–0.002 Gt C^54^ and shells that are one of the most important constituents of inorganic carbon flux^22, 21^. An overall decrease in foraminiferal calcite under low pH conditions could potentially impact the amount of calcite both contributed to the alkalinity pump and available for “ballast”, although this later outcome remains difficult to quantify^55^. As both respiration and spine repair were significantly inhibited under low [CO_3_^2−^] conditions, there is also potential for greater non-reproductive mortality in *G*. *bulloides* under future low [CO_3_^2−^] scenarios, which could increase export of organic carbon within the cytoplasm from surface waters by means of a rapidly sinking shell (29–552 m day^−1^)^48^.

Increased export of foraminiferal soft tissue, a constituent of particulate organic carbon (POC) flux, would generally intensify the biological pump and the drawdown of CO_2_ into the deep sea. In parallel, sinking foraminifera shells would be less calcified, resulting in less CaCO_3_ precipitated and then exported out of surface waters, reducing the foraminiferal contribution to the alkalinity pump. The opposing roles of the biologic and alkalinity pumps in ocean carbon cycling can be summarized by the “rain ratio” of CaCO_3_/POC of particles exported to the sediment, which represents the strength of the biologic pump relative to the alkalinity pump. Our results suggest that foraminiferal responses to ocean acidification will result in a decreased rain ratio of CaCO_3_/POC, by a more efficient export of organic carbon out of the surface ocean relative to the counterbalancing effect of the drawdown of alkalinity due to calcification. Such a decrease in the rain ratio could represent a negative climate feedback to ocean acidification^21–23^, exporting more organic carbon than alkalinity. The simultaneous response of both reduced calcification and increased organic carbon export by foraminifera at low pH would strengthen the efficiency of CO_2_ drawdown by the biologic pump. Therefore, in areas in which foraminifera are major contributors to carbon flux, stress-induced changes to foraminiferal CaCO_3_/POC at pH < 7.7 could provide a significant negative feedback on near-surface acidification and climate change.

For a pH drop from a baseline of 8.0 to a future condition of 7.6 units, we estimate a corresponding decrease in the rain ratio, based on our observations of reduced foraminifera calcification and likely increased cytoplasm export associated with spine loss at low pH (independent of other biological consequences of ocean acidification). We use previously published records of foraminiferal CaCO_3_, total CaCO_3_, and POC flux below 1000 m (Table 1). For this estimate, we assume that an average individual foraminifera contains 5 μg CaCO_3_, based on sediment trap total weights and counts of individual foraminifera, and 1.7 μg POC, based on observations of a global average CaCO_3_ to POC ratio of 3:1 in planktic foraminifera^56–61^. We furthermore assume that our calcification results are typical for all foraminifera at pH < 8.0, that species composition and therefore biomass production remains constant, and that absence of spine repair is a reasonable predictor of increased non-reproductive mortality. Under these circumstances, a reduction in foraminiferal calcification would decrease total CaCO_3_ flux by 12–30%, and increase POC flux by 5–11%. This results in a decrease of the global rain ratio of 15–38% due to changes in foraminifera calcification and physiology alone, with the primary uncertainty being the foraminiferal contribution to global CaCO_3_ flux^22^. This same set of assumptions can be applied to single locales in which carbon fluxes and foraminifera contribution are better constrained (Table 1).

**Table 1:** Potential changes in foraminiferal contribution to CaCO_3_ and POC flux, and the ‘rain ratio’.

Location	Average Foraminifera Flux (μg m^−1^ day^−1^)	Average CaCO_3_ Flux (μg m^−1^ day^−1^)	Average POC Flux (μg m^−1^ day^−1^)	Change in CaCO_3_ Flux (%)	Change in POC Flux (%)	Change in Rain Ratio (%)
Santa Barbara Basin (Coastal Upwelling)	6350^57^	181000^58^	96000^58^	1%	<1%	2%
Station Papa (Open Ocean)	12110^59^	12000^60^	15000^60^	38%	9%	43%
Cariaco Basin (Equatorial Upwelling)	14495^61^	37000^58^	34000^58^	15%	5%	19%
Global Average	0.36 Gt/y-0.88 Gt/y^21^	1.1 Gt/y^62^	0.86 Gt/y^63^	12–30%	5–11%	15–38%

Fluxes are taken from the deepest available published sediment-trap measurements at each site for Santa Barbara Basin (500 m) in the Southern California Current System, Station Papa (1000 m) in the North Pacific, and Cariaco Basin (1200 m) in the tropical Atlantic^56–61^. Global averages assume a depth of >1000 m and are based on a range of potential contributions of foraminifera calcite to CaCO_3_ flux at depth^21, 61–63^.

The observed reduction in calcification with possible metabolic depression in response to reduced [CO_3_^2−^] also suggests a potential for ambiguity in the fossil record. Although some studies have shown more complexity in the field than in the tightly coupled weight:[CO_3_^2−^] relationships seen in culture^20, 31^, no field studies of foraminiferal calcification have been conducted at [CO_3_^2−^] below modern open ocean values or the extremes at which corresponding metabolic depression and reduced spine repair are here observed. Thus, there may be a limit to the ability of foraminiferal calcification to compensate for [CO_3_^2−^] variability occurring in some natural systems. As *G*. *bulloides* calcifies less under low [CO_3_^2−^], they may record fewer extreme low [CO_3_^2−^] environmental conditions, such as upwelling events, that occur within a foraminiferal lifetime (~4 weeks). This may pose little problem for reconstructions of long-term acidification events in the open ocean due to relatively slow rates of change and the potential for adaptation. However, bias against calcification at reduced [CO_3_^2−^] could be important in more variable environments, where foraminifera may respond to low [CO_3_^2−^] events with a temporary reduction in calcification and metabolic rate, or increased mortality. Thus, low [CO_3_^2−^] conditions may be underrepresented in the fossil record.

This study emphasizes the biological and physiological consequences of pH extremes, even in short exposures, for planktic foraminifera. While the California Current and other eastern boundary current upwelling regions are among the first to experience large departures from global-average values of ocean pH, downward shifting baselines and new pH minima are likely to be encountered in an increasing number of environments over the next century^39, 40^. In upwelling regions, pH is already seasonally comparable to the lowest pH treatments tested here, and the upwelling seasons can also coincide with the greatest flux of *G*. *bulloides* and other planktic foraminifera^36, 57^. The potential for changes originating in low-trophic marine calcifiers, such as planktic foraminifera, to impact regional and global carbon cycling and ecosystems is therefore of increasing concern and relevance for the future ocean.

## Electronic supplementary material

Supporting Information
