# Supplementary material for: Ocean acidification compromises a planktic calcifier with implications for global carbon cycling
Source: Sci Rep. 2017 May 22;7:2225. doi: 10.1038/s41598-017-01530-9 (PMC5440396; doi:10.1038/s41598-017-01530-9)
Supplement: Supplementary file 1 — Supporting Information [file 41598_2017_1530_MOESM1_ESM.pdf]

Full title: Ocean acidification compromises a planktic calcifier with implications for global carbon cycling: Supplemental Material

Authors: Catherine V. Davis[a,b,*], Emily B. Rivest[b,c], Tessa M. Hill[a,b], Brian Gaylord[b, d], Ann D. Russell[a], Eric Sanford[b,d]

Address:
[a]Department of Earth & Planetary Sciences, University of California - Davis, Davis, CA, USA
[b]Bodega Marine Laboratory, University of California - Davis, Bodega Bay, CA, USA
[c]Present Address: Virginia Institute of Marine Science, William & Mary, Gloucester Point, VA, USA
[d]Department of Evolution and Ecology, University of California - Davis, Davis, CA, USA

[*] Email: cvdavis@seoe.sc.edu; School of the Earth, Ocean and Environment, 701 Sumter St., Columbia, SC, 29208, USA; (803) 777-4588

Experiment Run	Surface Conditions at Collection			Conditions during Incubation for Oxygen Consumption			Average Conditions for Entire Experiment			
	pH_T	$[CO_3^{2-}]$ (μmol kg^{-1})	Ω_{Ca}	pH_T	$[CO_3^{2-}]$ (μmol kg^{-1})	Ω_{Ca}	pH_T	$[CO_3^{2-}]$ (μmol kg^{-1})	Ω_{Ca}	Salinity (psu)
C1	8.08	132.55	3.47	7.59	57.09	1.31	7.56	53.8	1.39	32.5
C2	8.08	132.55	3.47	8.15	179.65	4.40	8.16	180.97	4.36	32.5
D1	8.18	174.23	4.34	7.75	79.71	1.82	7.72	75.08	1.93	32.98
D2	8.18	174.23	4.34	8.27	221.89	5.30	8.27	219.18	5.36	32.98
E1	8.14	165.08	4.01	7.53	49.76	1.21	7.59	56.78	1.38	32.54
E2	8.14	165.08	4.01	7.75	80.06	1.68	7.69	69.24	1.94	32.54
F1	8.05	139.28	3.38	7.97	117.70	2.90	7.96	119.6	2.86	32.46
F2	8.05	139.28	3.38	8.28	224.22	5.40	8.28	222.58	5.44	32.46
G1	8.05	141.73	3.42	7.72	70.95	1.56	7.66	64.03	1.72	32.19
G2	8.05	141.73	3.42	8.00	129.18	3.04	7.98	125.19	3.14	32.19
H1	8.04	137.87	3.30	7.59	55.69	1.18	7.52	48.47	1.35	32.54

Table S1. Carbonate chemistry parameters for each experimental run, including sea surface parameters at the time of collection, measurements of treatment water at the time of incubation for respirometry, and average conditions (from water samples taken every other day) over the entire experimental run. Each letter represents a single collection and letter and number pair an experimental run.

Experimental Group	pH Before Filling	pH After Filling	$[CO_3^{2-}]$ (μmol/kgSW) Before Filling	$[CO_3^{2-}]$ (μmol/kgSW) After Filling
C1	7.59	7.59	56.70	57.48
C2	8.15	8.16	178.25	181.04
D1	7.73	7.76	77.22	82.20
D2	8.28	8.27	224.53	219.26
E1	7.57	7.61	54.61	58.95
E2	7.74	7.76	78.39	81.72
F1	7.98	7.96	119.28	116.13
F2	8.28	8.28	223.34	225.10
G1	7.69	7.74	67.17	74.73
G2	8.00	8.00	129.08	129.27
H1	7.58	7.60	54.90	56.49

Table S2. During respirometry incubations, individual foraminifera were maintained in treatment water without a fluorescent calcein label. Treatment water was filtered from 0.6μm to 0.2μm for this purpose and to account for any change in chemistry as a result of the filtration, pH (total scale) was measured before and after the vials were filled.

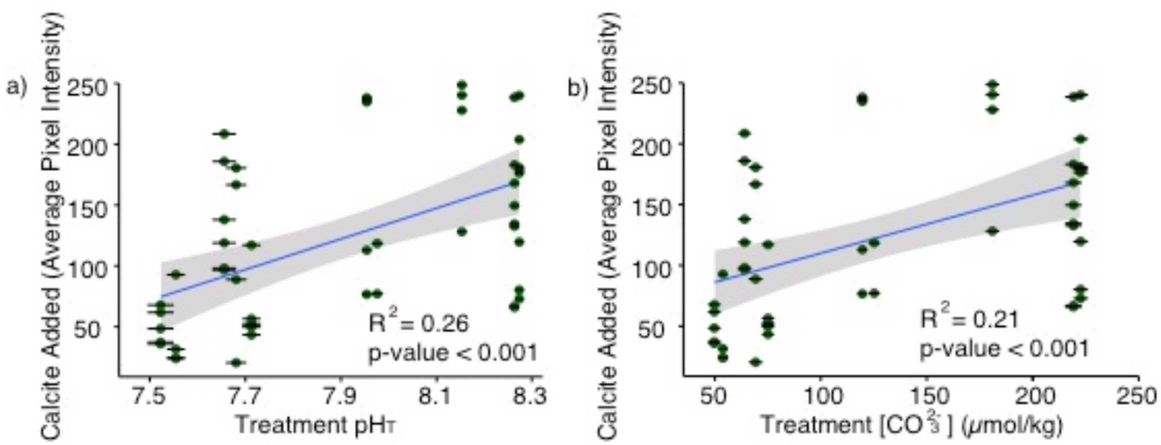

Fig S1. Individual foraminifera pixel intensity shows calcite added for single shells. Reduced calcification under low pH or [CO_3^{2-}] has previously been described for foraminifera in culture (1-3), in plankton tows (4), and in the fossil record (5, 6), and is generally assessed by a ratio of weight to size. The application of this methodology to foraminifera is novel, allowing for a semi-quantitative documentation of only the amount of calcite grown under known (culture) conditions. This is a useful approach in assessing calcification of individuals that did not add a new chamber under culture conditions.

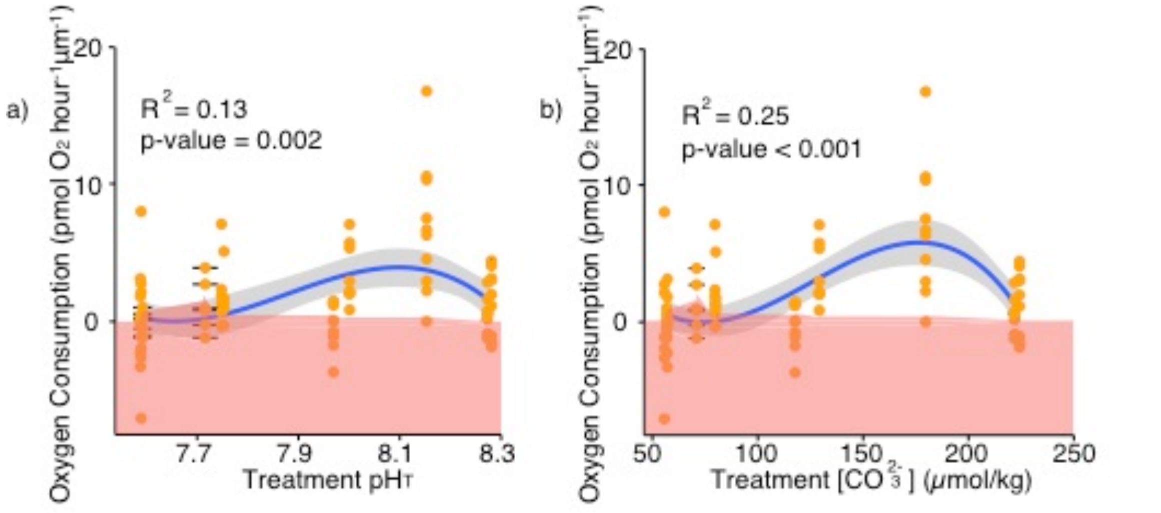

Fig. S2 Aerobic metabolism was assessed as the rate of oxygen consumption relative to background respiration in filtered seawater and normalized to shell length (pmol O_2 foram^{-1} hr^{-1} μm^{-1}). Individual foraminifera showed a large degree of inter-individual variability in respiration rates. The cause of this variability is indicative of differing physiologic states across individual shells, not captured by quantitative or qualitative metrics of health or life history. Such internal processes must play an important and as of yet undefined role in foraminiferal oxygen consumption. The pink shaded region represents results indistinguishable from background.

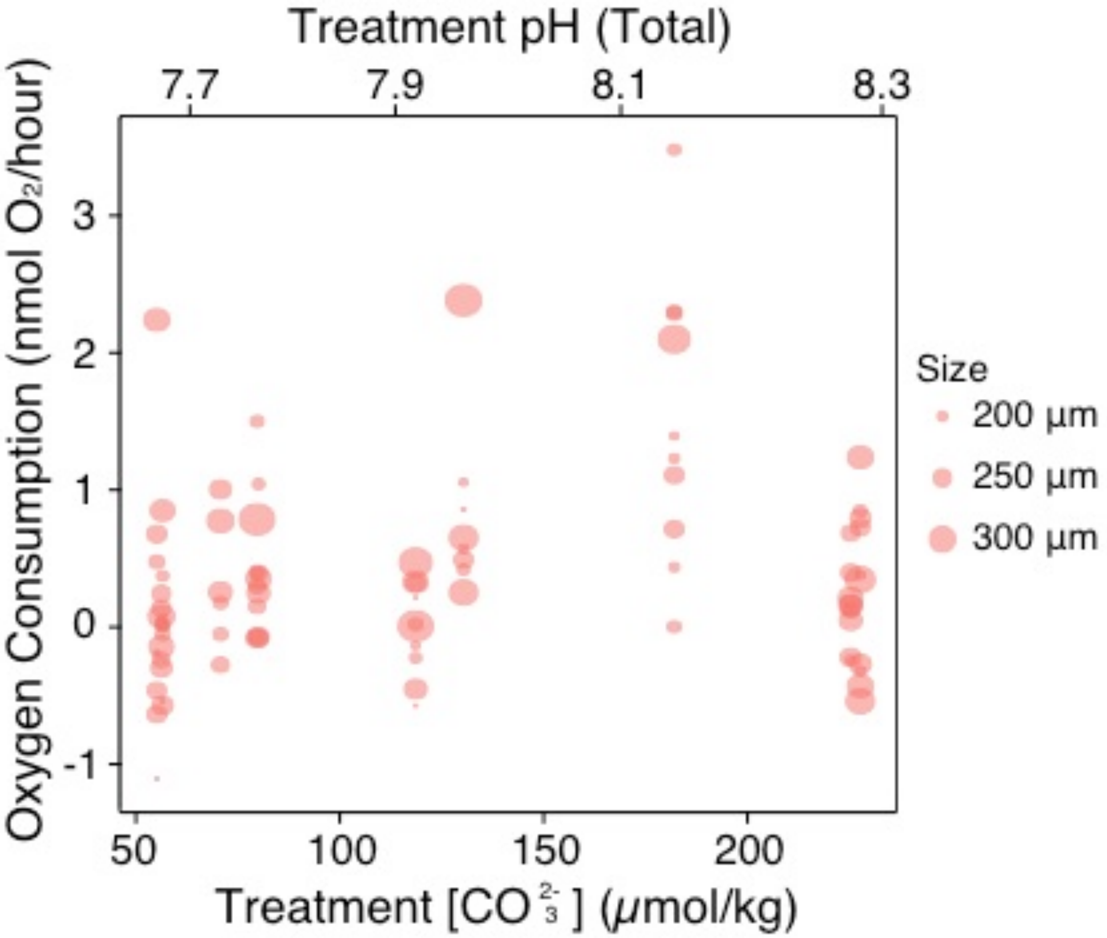

Fig S3. Oxygen consumption rates (nmol O_2 foram^{-1} hr^{-1}) across pH treatments, sized according to the longest shell dimension. Shell length has been shown to relate to biomass in both planktic (7) and benthic foraminifera (8, 9) and with protein biomass in planktic foraminifera (10), and as such, maximum shell length from post-respirometry observations was used to normalize oxygen consumption rates to account for variation associated with approximate biomass (pmol O_2 foram^{-1} hr^{-1} µm^{-1}). Here, un-normalized oxygen consumption rates (nmol O_2 foram^{-1} hr^{-1} µm^{-1}) are shown, sized according to the longest shell dimension.

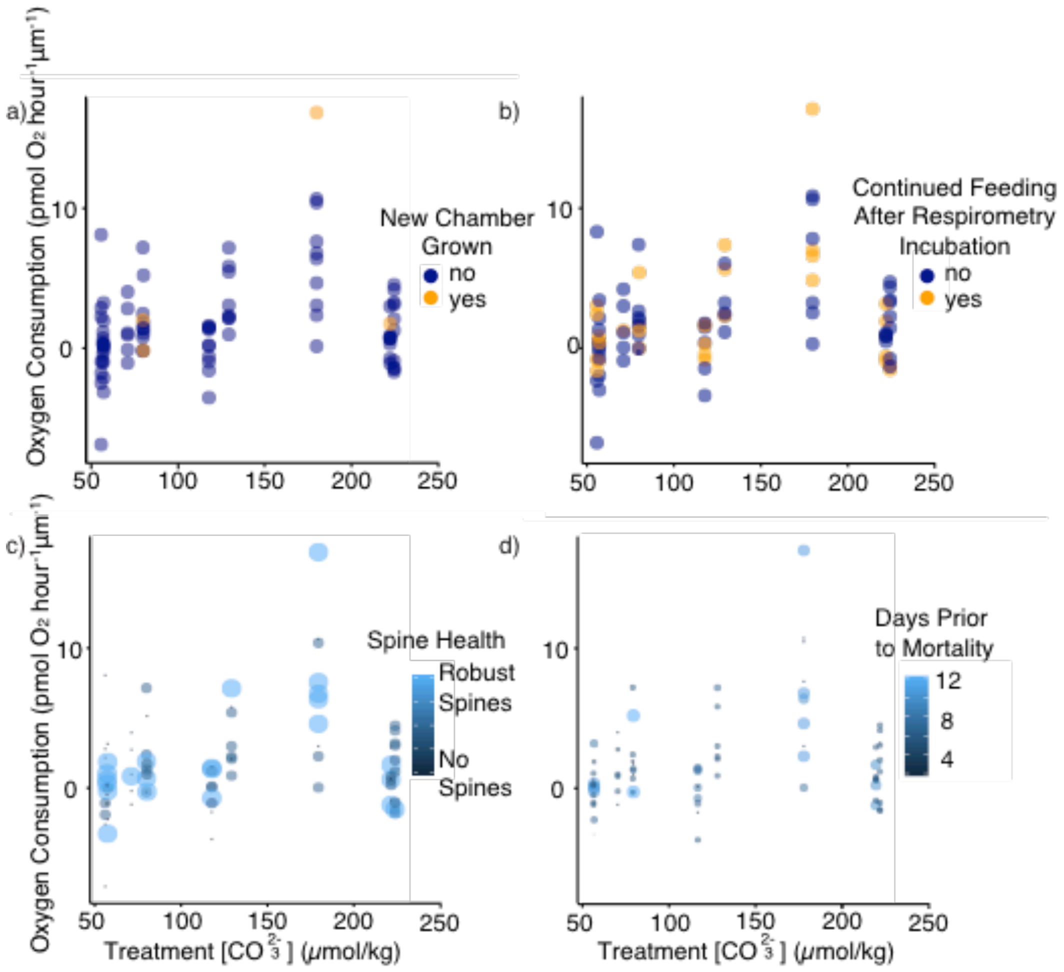

Fig. S4 The relationship between oxygen consumption and other observed traits in *G. bulloides* relative to [CO_3^{2-}]. (A) Individuals that added a new chamber during their respirometry incubation are highlighted in orange. (B) Individuals that were able to feed the day following respirometry incubation (thus both healthy and not pre-gametogenic) are highlighted in orange. (C) Individuals with an extensive spine array at the time of incubation are highlighted in lighter blue. (D) The number of total days in culture before death or gametogenesis, with individuals having longer culture lifespans in lighter blue.

Water Chemistry

Water for each treatment condition was prepared ahead of collection. Individual *G. bulloides* were immediately isolated from plankton tows and randomly assigned to a target pH treatment (pH ~ 7.5, 7.7, 8.0, or 8.3) prior to the initiation of experiments. Limited abundance in tow material prevented all four treatments from being run

simultaneously. Consequently, the experimental conditions were replicated in non-repeating pairs until all four targets had been run at least twice. At the start of each experiment, foraminifera were placed directly into 25 mL vials, sealed without headspace, and filled with treatment water filtered to 0.6 um containing fluorescent calcein dye. A regression of measured conditions was used in most analyses, through, to assess such categorical traits (i.e. spine regrowth, chamber addition), the proportion of individuals with a trait are reported relative to the measured average condition within each of four pH groups, averaging pH_T 7.54 (SE +/- 0.02), 7.69 (SE +/- 0.01), 8.02 (SE +/- 0.02) and 8.27 (SE +/- 0.01), respectively.

To monitor carbonate chemistry conditions throughout each run, a new container of treatment water was opened for each water change and measurements of pH_T and total alkalinity were performed both upon opening and after filling vials. pH_T was measured using a spectrophotometric method based on *m*-cresol purple (10) (UV-1800 UV-VIS spectrophotometer, Shimadzu Corp., Kyoto, Japan) (+/- 0.03 pH_T). Total alkalinity was measured using an automated, open-cell potentiometric titration (12) with a Metrohm 809 Titrando titrator and a Metrohm 6.0262.100 pH probe (Metrohm AG, Herisau, Switzerland) (+/- 4.2 umol/L). Titrations were performed using a titrant of 0.025N HCl in a matrix of NaCl (33.5 PSU) and were standardized using certified references materials from A. Dickson laboratory (Scripps Institute of Oceanography). Temperature, salinity, pH_T, and total alkalinity were used to calculate the complete suite of carbonate chemistry parameters for each water change, using CO2CALC (13), with CO_2 constants K1, K2 (14) and pH_T (mol kg-SW^{-1}).

Defining Foraminiferal Life Stage

We used established understandings of foraminiferal life history to guide our observations. This included the important observation that the planktic foraminiferal life cycle ends in sexual reproduction and the release of gametes, with the parent cytoplasm being nearly or entirely consumed or converted in the process (15). Individuals were photographed every other day in culture and observed every day for the presence and robustness of spines, extended rhizopodia, cytoplasm color, and the degree to which cytoplasm filled the youngest chambers. Individual *G. bulloides* generally completed their life cycle within 3-10 days of collection. 1-2 days prior to gametogenesis, *G. bulloides* began to show “pre-gametogenic” characteristics, including change of cytoplasm color from brown/gold to white, rhizopodial shortening and decreased “streaming”, and cessation of feeding. In some cases foraminifera died without completing their life cycle and “death” was defined as the inability to feed during two consecutive feedings accompanied by an absence of rhizopodial activity without pre-gametogenic characteristics. Foraminifera that did not feed but appeared to be pre-gametogenic were subject to continued observations, imaging, and water changes until gamete release.

Incubation in Calcein

The fluorescent compound, calcein (Sigma-Aldrich, Co., St. Louis, MO, USA), was used to label foraminiferal calcite grown under treatment conditions. Calcein binds to free calcium ions and is incorporated in calcite minerals during shell formation from seawater.

Calcein was shown to be non-toxic to foraminifera during short (<5 week exposures) at concentrations <20 mg/L (16, 17) and has since been used in additional experiments e.g. (18, 19). All foraminifera were incubated in treatment seawater containing a final calcein concentration of 10 mg/L. Calcein addition was found to cause a small decrease on final seawater pH (< 0.02), which given the slightness of this change and consistency of calcein addition across treatments was not chemically corrected. When the number of days spent in culture, and therefore in calcein was included in initial statistical models of calcification relative to pH, there was no significant interaction between time and either carbonate chemistry parameter.

Analogous uses of calcien as a quantitative or semi-quantitative measurement of calcite bound calcite has been undertaken by many previous authors, for example as a quantitative metric of coral calcification (20) or semi-quantitatively for *in vitro* analyses (21). Results here are presented as semi-quantitative, as the relationship between calcification and calcein uptake under these conditions in foraminiferal calcite could not be quantified. Our semi-quantitative analysis of bound calcien was carried out using the Metamorph software, by defining the limits of the foraminiferal shell from images taken using an epifluorescent microscope. An upper and lower threshold of brightness consistent across all images was defined, and then the relative brightness or intensity of each pixel could be determined. The measure reported, of Average Pixel Intensity, was the average intensity of each pixel of our image defined as shell.

Predicting Changes in the 'Rain Ratio'

In our estimations of foraminfieral contribution to 'rain ratio' changes, we used previously published records of foraminiferal $CaCO_3$, total $CaCO_3$, and POC flux below 1000 m (Table 2), along with an approximation that each individual foraminifera contains on average 5 μg $CaCO_3$ and 1 μg POC. We furthermore assumed that our calcification reduction results are representative of all foraminifera at pH <8.0, and that absence of spine repair is a reasonable measure of pre-reproductive mortality and rapid export. Thus, foraminifera shells would contain on average 38% less $CaCO_3$. If a spine loss event occurs (we estimate this at 25% likelihood within a lifetime), 70% of foraminifera would fail to recover, resulting in export of POC associated with cytoplasm. We also assume no change in "background" mortality, such as that of thinly calcified sub-adults, for which remobilization of both organic and inorganic carbon likely occurs in the upper water column, and thus would not impact export.

References

1. Bijma J, Spero H, & Lea D (1999) Reassessing foraminiferal stable isotope geochemistry: Impact of the oceanic carbonate system (experimental results). *Use of proxies in paleoceanography*, (Springer), pp 489-512.
2. Manno C, Morata N, & Bellerby R (2012) Effect of ocean acidification and temperature increase on the planktonic foraminifer *Neogloboquadrina pachyderma* (sinistral). *Polar Biol* 35(9):1311-1319.
3. Russell AD, Hönisch B, Spero HJ, & Lea DW (2004) Effects of seawater carbonate ion concentration and temperature on shell U, Mg, and Sr in cultured planktonic foraminifera. *Geochimica et Cosmochimica Acta* 68(21):4347-4361.
4. Marshall BJ, Thunell RC, Henehan MJ, Astor Y, & Wejnert KE (2013) Planktonic foraminiferal area density as a proxy for carbonate ion concentration: A calibration study using the Cariaco Basin Ocean Time Series. *Paleoceanography*:n/a-n/a.
5. Barker S & Elderfield H (2002) Foraminiferal Calcification Response to Glacial-Interglacial Changes in Atmospheric CO_2. *Science* 297(5582):833-836.
6. Moy AD, Howard WR, Bray SG, & Trull TW (2009) Reduced calcification in modern Southern Ocean planktonic foraminifera. *Nature Geosci* 2(4):276-280.

7. Schiebel, R. & Movellan, A. (2012) First-order estimate of the planktic foraminifer biomass in the modern ocean. *Earth Syst. Sci. Data* 4:75-89.
8. Altenbach AV (1987) The measurement of organic carbon in foraminifera. *The Journal of Foraminiferal Research* 17(2):106-109.
9. Movellan, A., Schiebel, R., Zubkov, M. V., Smyth, A., & Howa, H. (2012) Protein biomass quantification of unbroken individual foraminfiers using nano-spectrophotometry. *Biogeosciences* 9:3613-3623.
10. Fenchel T & Finlay B (1983) Respiration rates in heterotrophic, free-living protozoa. *Microbial Ecology* 9(2):99-122.
11. Meilland, J, Hele, H, Monaco, CL, & Schiebel, R (2016) Individual planktic foraminifer protein-biomass affected by trophic conditions in the Southwest Indian Ocean, 30°S-60°S. *Mar Micropal* 124: 63-74.
12. Dickson AG, Sabine CL, & Christian JR (2007) Guide to Best Practices for Ocean CO_2 Measurements.
13. Robbins LL, Hansen, M.E., Kleypas, J.A., and Meylan, S.C. (2010) CO2calc: A user-friendly seawater carbon calculator for Windows, Max OS X, and iOS (iPhone). *U.S. Geological Survey Open-File Report 2010-1280*.
14. Lueker TJ, Dickson AG, & Keeling CD (2000) Ocean pCO_2 calculated from dissolved inorganic carbon, alkalinity, and equations for K1 and K2: validation based on laboratory measurements of CO_2 in gas and seawater at equilibrium. *Marine Chemistry* 70(1):105-119.
15. Hemleben C, Spindler M, & Anderson OR (2012) *Modern planktonic foraminifera* (Springer Science & Business Media).
16. Bernhard JM, Blanks JK, Hintz CJ, & Chandler GT (2004) Use of the fluorescent calcite marker calcein to label foraminiferal tests. *The Journal of Foraminiferal Research* 34(2):96-101.
17. Kurtarkar SR*, et al.* (2015) Assessing the effect of calcein incorporation on physiological processes of benthic foraminifera. *Marine Micropaleontology* 114:36-45.
18. Dissard D, Nehrke G, Reichart GJ, & Bijma J (2010) Impact of seawater pCO_2 on calcification and Mg/Ca and Sr/Ca ratios in benthic foraminifera calcite: results from culturing experiments with Ammonia tepida. *Biogeosciences* 7(1):81-93.
19. Raitzsch M, Dueñas-Bohórquez A, Reichart G-J, de Nooijer LJ, & Bickert T (2010) Incorporation of Mg and Sr in calcite of cultured benthic foraminifera: impact of calcium concentration and associated calcite saturation state. *Biogeosciences* 7(3):869-881.
20. Hale, L. V., Ma, Y. F. & Santerre, R. F. (2000) Semiquantitative fluorescence analysis of calcein binding as a measurement of in vitro mineralization. *Calcif. Tissue Int.* 67:80–84.
21. Tambutte, E., Tambutte, S., Segonds, N., Zoccola, D., Venn, A., Erez, J. & Allemand, D. (2012) Calcein labelling and electrophysiology: insights on coral tissue permeability and calcification. *Proc. R. Soc. B.*279:19-27.
